# Use of hand hydraulic dynamometers as an overall evaluation of the upper-limb weakness in patients with thoracic outlet syndrome

**DOI:** 10.3389/fneur.2022.919312

**Published:** 2022-08-15

**Authors:** Alban Fouasson-Chailloux, Pauline Daley, Pierre Menu, Guillaume Gadbled, Yves Bouju, Giovanni Gautier, Germain Pomares, Marc Dauty

**Affiliations:** ^1^Service de Médecine Physique et Réadapatation Locomotrice et Respiratoire, CHU Nantes, Nantes Université, Nantes, France; ^2^Service de Médecine du Sport, CHU Nantes, Nantes Université, Nantes, France; ^3^IRMS, Institut Régional de Médecine du Sport, Nantes, France; ^4^Inserm, UMR 1229, RMeS, Regenerative Medicine and Skeleton, Nantes Université, ONIRIS, Nantes, France; ^5^Clinique Chirurgicale Orthopédique et Traumatologique, CHU Nantes, Nantes Université, Nantes, France; ^6^Institut Main Atlantique, Saint Herblain, France; ^7^Explorations Fonctionnelles Vasculaires, CHU Nantes, Nantes Université, Nantes, France; ^8^Institut Européen de la Main, Hopital Kirchberg, Luxembourg, Luxembourg

**Keywords:** isokinetic, grip, shoulder, strength, neurogenic thoracic outlet syndrome

## Abstract

Patients with neurogenic thoracic outlet syndrome report pain and upper-limb weakness. They complain about weakness occurring on the entire upper-limb, especially at the hand and the shoulder levels. Hydraulic dynamometers can reliably assess the strength of the hand, and isokinetic shoulder testing can provide accurate and reliable evaluations of the rotators strength. Yet, isokinetic proximal assessment needs expensive tools, whereas hydraulic hand dynamometers are cheap and easy to use. We aimed to assess the correlation between the isokinetic shoulder strength and the hand grip and the key pinch strength. The grip strength was evaluated with a hydraulic hand dynamometer and the key pinch with a pinch gauge. Isokinetic rotators strength tests were performed using a Humac Norm® dynamometer at 60 and 180°/s. One-hundred and thirty patients had been included, 72% of women, mean age of 39.8 ± 9.5. Symptomatic hands presented a strength deficit of 12.2% on the grip (*p* < 0.0001) and 10% on the key pinch (*p* = 0.01). Isokinetic strength was lower on the symptomatic shoulders at 60 and 180°/s concerning medial rotators [−10.3 and −8.8%, respectively (*p* = 0.02)] and lateral rotators [−10.8 and −10%, respectively (*p* = 0.04 and *p* = 0.03)]. There was a moderate correlation between the grip strength of the symptomatic upper-limbs and the isokinetic rotators strength (*p* < 0.001). The key pinch strength was moderately correlated to the isokinetic medial and lateral rotators strength at 60°/s (*p* < 0.001). Hand dynamometers could prove useful during medical consultations or in outpatient management to assess upper-limb overall weakness, but isokinetic measurement remains the gold standard for a precise evaluation.

## Introduction

Thoracic outlet syndrome (TOS) is a relatively rare entity (1, 2) which includes all the manifestations due to the compression of the upper-limb neurovascular bundle (3). Three forms exist depending on the involved anatomic structures: neurogenic TOS (NTOS), venous TOS and arterial TOS (4, 5). NTOS represents about 90% of the cases (3, 4, 6), resulting from the association of anatomic predispositions and various local factors responsible for intermittent compression of the brachial plexus at the supraclavicular scalene triangle and/or at the sub-coracoid space levels (6–8). Patients with NTOS usually report chronic pain responsible for functional disability and social and professional impairment (7, 9, 10). Women are more frequently affected at around 40 years old (7, 11). Main symptoms are upper-limb pain, paresthesia and weakness, especially during prolonged elevated arm position or during repetitive upper-limb movements. NTOS remains a challenging diagnosis (5, 7, 12–14). Yet, recent guidelines allow an easier diagnosis (5, 7, 15, 16).

Weakness and strength deficit are mostly reported by patients with NTOS, and they are also considered diagnostic criteria (5, 7, 15, 17–19). Besides, patients usually complain of a strength deficit occurring on the entire upper-limb, especially at the hand and the shoulder levels (18, 19). Indeed, Fouasson-Chailloux et al. (18) recently confirmed a significant hand strength deficit both on the symptomatic and the asymptomatic hands. Likewise, Daley et al. (19) also showed a severe isokinetic strength deficit of the medial and lateral shoulder rotators. Thereby, these two studies highlighted the interest of proximal and distal upper-limb strength measurement in the evaluation of NTOS (18, 19). In fact, hydraulic hand dynamometers and pinch gauges can reliably assess strength at the hand level (20–22), and isokinetic shoulder testing can provide accurate and reliable evaluations to measure shoulder strength, especially for medial and lateral rotator muscles (23–25). However, while distal measurement is easy, isokinetic proximal assessment needs expensive tools, experienced practitioners and time. Indeed, hydraulic hand dynamometers and pinch gauges are rather cheap devices and easy to use, whereas isokinetic dynamometers are expensive and most often only accessible in hospital settings (26). Interestingly, some publications have suggested a link between grip strength and isokinetic shoulder strength, with Pearson correlations from 0.40 to 0.82 (27–29). Yet, it concerned small groups of participants and no studies have assessed the particular context of NTOS.

In this study, we aimed to assess the correlation between isokinetic strength of the shoulder rotators and, the hand grip and the key pinch strength in order to know if hydraulic dynamometers could be used routinely as an overall evaluation of the upper-limb weakness.

## Materials and methods

### Patients

All the patients with NTOS were recruited from July 21^st^, 2020, to February 7^th^, 2022. They were assessed at the beginning of an inpatient protocol of rehabilitation. This rehabilitation program concerned patients in the case of ineffective outpatient physiotherapy of at least 6 months. The rehabilitation program consisted in a 3- to 4-week period. To be included, patients had to fulfill the diagnostic criteria for NTOS according to the Consortium for Research and Education on thoracic outlet syndrome (7, 15) and to be over 18. Exclusion criteria were: (1) upper-limb musculoskeletal disorders (rotator-cuff tendinopathy, osteoarthritis for example), (2) cervical-brachial neuralgia, (3) other entrapment neuropathy of the upper limb [all the patients with NTOS underwent electro-diagnostic testing before inclusion (30)], (4) and any contraindication to the isokinetic testing. After inclusion, patients' pain was evaluated with a numerical rating scale (NRS) ranging from 0 to 10 (31). They also completed a French version of the QuickDASH (32), evaluating upper-limb function and symptoms. The score ranges from 0 (no disability) to 100 (most severe disability), and has frequently been used in the context of NTOS (18, 19, 33–35). All the patients performed a strength assessment, including shoulder isokinetic testing of the rotator cuff and hand-grip and key-pinch measurement.

This study is part of a protocol approved by the Committee of Ethics “Comité de Protection des Personnes d'Ile-de-France II,” and all the patients gave their verbal consent to participate in the study. No written consent was needed for the participants because the study did not modify patients' usual care. The study was registered on ClinicalTrials.gov: NCT04145778.

### Isokinetic testing

The tests were performed as previously described (19). Briefly, after a 5 min warm up with an arm cranking ergometer (Ergoselect® 400, Ergoline, Bitz, Germany), isokinetic strength tests were performed using a Humac Norm® dynamometer (CSMI, Stoughton, MA, USA). The medial and lateral rotations were performed in the scapular plane in sitting position. The amplitudes of the medial and lateral rotations of the shoulder were fixed at 65 and 35°, respectively. In the case of unilateral NTOS, the non-symptomatic shoulder was tested first. The two shoulders were evaluated in a random order in case of bilateral NTOS. After familiarization with the isokinetic movement (5 sub-maximal repetitions at 240°/s), the subjects were tested with 3 repetitions of concentric medial and lateral shoulder rotations at 60°/s, followed by 5 repetitions at 180°/s. Recovery between the series was 20s. Two isokinetic parameters were taken into consideration: relative peak torque defined by the maximal peak torque normalized to the body mass (Nm/kg) at the 2 angular speeds (60°/s and 180°/s). The reliability previously established by intra-class correlation coefficient (ICC) of the concentric isokinetic peak torque at 60°/s is excellent on medial rotation (ICC: 0.93 on left side; 0.94 on right side) and lateral rotation (ICC: 0.92 on left side; 0.95 on right side) (24). The reliability of the concentric isokinetic peak torque at 180°/s is good to excellent (ICC medial rotation: 0.98 on left side; 0.97 on right side; ICC lateral rotation: 0.91 on left side; 0.92 on right side) (19).

### Grip and key pinch strength testing

Subjects were seated in a standardized position as previously described (18), with their shoulder adducted and neutrally rotated, elbow flexed at 90°, with the forearm and the wrist in neutral position. The two hands were evaluated in a random order in case of bilateral NTOS, and in case of unilateral NTOS, the non-symptomatic upper limb was tested first. Grip strength was evaluated with a hydraulic hand dynamometer (Baseline®, Irvington, NY 10533, USA) and the key pinch with a pinch gauge (Baseline®, Irvington, NY 10533, USA), which have excellent reliability (ICC from 0.94 to 0.95 and ICC from 0.83 to 0.91, respectively) (18, 22).

### Statistical analysis

Statistical analyses were performed with IBM SPSS 23.0 software (Armonk, NY, USA). Quantitative parameters were presented as mean and standard deviation. The Kolmogorov–Smirnov test was used to assess the normality of the data. Taking the upper-limb as unit (36), quantitative variable comparisons between symptomatic upper-limbs and asymptomatic upper-limbs were performed with Mann–Whitney tests (data were not normally distributed), and qualitative comparisons were performed with χ^2^ tests. Spearman's correlation coefficients (*R*^2^) were calculated to assess the association between the isokinetic shoulder strength parameters and the hand strength parameters. The correlation coefficient interpretation was (37): strong correlation (*R*^2^ > 0.9); high (0.7 < *R*^2^ < 0.9); moderate (0.5 < *R*^2^ < 0.7), low (0.3 < *R*^2^ < 0.5), negligible (*R*^2^ < 0.3). The level of significance was considered at *p* < 0.05.

## Results

### Patients' characteristics

One-hundred and thirty patients with NTOS were included, 94 women (72%) and 36 men (28%), mean age was 39.8 ± 9.5. Their mean height was 167.5 ± 8.0 cm, mean weight 71.7 ± 16.4 kg, and mean Body Mass Index (BMI) 25.4 ± 4.9. Eighty-four patients had unilateral NTOS and 46 had a bilateral form. The mean duration of the symptoms was 3.0 ± 2.7 years. Patients had a mean QuickDASH score of 59.2 ± 13.9 and a mean pain on the NRS of 5.6 ± 1.7.

### Strength assessment of the upper-limbs

Symptomatic hands presented a strength deficit of 12.2% on the grip (*p* < 0.0001) and 10.0% on the key pinch (*p* = 0.01) compared to asymptomatic hands (Table 1). Isokinetic strength was lower on the symptomatic shoulders both at 60°/s and 180°/s concerning medial rotators [-10.3 and−8.8%, respectively (*p* = 0.02)] and lateral rotators [-10.8 and−10.0%, respectively (*p* = 0.04 and *p* = 0.03)]. There was a higher proportion of dominant sides in the symptomatic upper-limbs (55.7%) compared to the asymptomatic ones (38.1%) (*p* = 0.008).

**Table 1:** Comparison of the strength between symptomatic and asymptomatic upper-limbs of patients with thoracic outlet syndrome (taking the upper-limb as unit).

	**Symptomatic upper-limbs, *n* = 176**	**Asymptomatic upper-limbs, *n* = 84**	**%StDef**	***P-*value**
Dom / non-Dom	98 / 78	32 / 52		0.008^#^
Grip, kg ± SD	25.1 ± 12.3	30.7 ± 10.7	−12.2%	<0.0001
Key pinch, kg ± SD	7.2 ± 2.5	8.0 ± 2.2	−10.0%	0.01
MR60, Nm ± SD	31.20 ± 12.4	34.8 ± 12.6	−10.3%	0.02
LR60, Nm ± SD	14.0 ± 6.7	15.7 ± 7.12	−10.8%	0.04
MR180, Nm ± SD	27.9 ± 11.5	30.6 ± 10.9	−8.8%	0.02
LR180, Nm ± SD	13.6 ± 5.0	15.1 ± 5.6	−10.0%	0.03

*Dom, dominant; non-Dom, non-dominant; SD, standard deviation; MR60, medial rotators at 60°/s; LR60, lateral rotators at 60°/s; MR180, medial rotators at 180°/s; LR180, lateral rotators at 180°/s; %StDef, percentage of strength deficit of the symptomatic upper-limbs compared to the asymptomatic upper-limbs. #: khi-2 tests*.

### Correlations between symptomatic hands and shoulders

There was a moderate correlation between grip strength of the symptomatic upper-limbs and, isokinetic medial and lateral rotators strength, both at 60 and 180°/s (r from 0.559 to 0.679, *p* < 0.001) (Table 2). Key pinch strength was moderately correlated to isokinetic medial and lateral rotators strength at 60°/s [r = 0.576 and r 0.569, respectively (*p* < 0.001)], and was lowly correlated to isokinetic medial and lateral rotators strength at 180°/s [r = 0.485 and r = 0.490, respectively (*p* < 0.001)].

**Table 2:** Correlations between isometric hand strength and shoulder isokinetic strength on symptomatic upper-limbs in patients with thoracic outlet syndrome.

	**MR60**	**LR60**	**MR180**	**LR180**
**Symptomatic upper-limbs**				
Grip	0.676***	0.660***	0.559***	0.578***
Key pinch	0.576***	0.596***	0.485***	0.490**
**Asymptomatic upper-limbs**				
Grip	0.610***	0.647***	0.497***	0.401***
Key pinch	0.535***	0.583***	0.538***	0.478***

MR60, medial rotators at 60°/s; LR60, lateral rotators at 60°/s; MR180, medial rotators at 180°/s; LR180, lateral rotators at 180°/s; ^***^p < 0.001; ^**^p < 0.01.

## Discussion

In this study, we aimed to assess the correlation between proximal and distal upper-limb deficit, so as to propose clinical practice advice. In fact, upper-limb weakness is one of the main symptoms reported by patients with NTOS, which has been objectively described both at the hand level and the shoulder level (18, 19). We highlighted a significant correlation between hand strength and shoulder rotators in a typical population of patients with NTOS. Indeed, our group of patients had usual characteristics of NTOS with a women rate of 72% and an age under 40 years old (38, 39). Moreover, the patients' impairment with a mean QuickDASH score of 59.2 ± 13.9 was quite consistent with previous studies (33, 34, 40).

As previously published, we confirmed a grip strength deficit on the symptomatic hand of about 12% compared to the asymptomatic side (18). We also found a deficit of strength on the key pinch and on the shoulder rotators on the symptomatic upper-limbs, whereas this was not the case in previous works which had found only significant decreases of strength compared to controls but no significant differences between symptomatic and asymptomatic sides, excepted concerning lateral rotators endurance (18, 19). The differences in the current study may be due to the greater number of included subjects from +30 to +53%, which can account for a stronger statistical power.

Interestingly, we found a moderate correlation between grip strength and isokinetic shoulder strength both at 60 and 180°/s on the symptomatic upper-limbs (from *r* = 0.578 to *r* = 0.676; p < 0.001). We also reported a moderate correlation between key pinch and isokinetic shoulder strength at 60°/s (from *r* = 0.576 to *r* = 0.596; *p* < 0.001), and a low correlation between key pinch and isokinetic shoulder strength at 180°/s (from *r* = 0.485 to *r* = 0.490; *p* < 0.001). Concerning, the asymptomatic sides, we also confirmed low to moderate correlation between proximal and distal upper-limb strength (from *r* = 0.401 to *r* = 0.647; *p* < 0.001). Yet, correlation between hand strength and isokinetic shoulder rotators strength remains arguable. In fact, in 18 healthy collegiate male athletes, Mandalidis and O'Brien (29) found only significant low to moderate correlations between hand grip and lateral rotators (from *r* = 0.40 to r = 0.54; *p* < 0.05), whereas no correlation was reported with medial rotators. In the same way, Ahmadi et al. (27) studied 12 elite sitting volleyball players with no upper-limb impairment. They showed moderate to high correlations between hand grip strength and isokinetic lateral rotator peak torques at 60°/s (from r = 0.67 to r = 0.72; *p* < 0.05), but also an absence of correlation with medial rotators. On the contrary, Nascimento et al. (28) assessed 12 patients with chronic hemiparesis and reported moderate to high significant correlations between isometric hand grip and isokinetic shoulder strength on medial and lateral rotators at 60°/s, both on the hemi-paretic side and the non-paretic side (from *r* = 0.61 to *r* = 0.82; *p* < 0.05). Thus, our results may have a practical impact, as grip and key pinch strength are easily measurable, they could provide an overall estimate of the upper-limb strength, including at the proximal level. This could prove useful during medical consultations or in outpatient management of patients. Yet, we believe that the correlations remain insufficient for an evaluation prior surgery or a hospital rehabilitation protocol for which an accurate and reliable assessment is necessary. In these last cases, we still recommend the use of isokinetic devices (19). An alternative to isokinetic testing could be the use of shoulder handheld dynamometers, which are a low-cost and portable method (41), but their evaluations remain to be done in NTOS. Moreover, other types of upper-limb assessment such as supination/pronation could be interesting in these patients. Yet, their measurements have few descriptions in the literature, which make their applicability and interpretation more difficult.

Our results showed a higher rate of dominant sides in the symptomatic upper-limbs (55.7%) compared to the non-symptomatic limbs (*p* = 0.008). We assume it has a negligible to a low impact on the interpretation of our results. Indeed, Madalidis and O'Brien (29) previously suggested an absence of strength difference between dominant and non-dominant upper-limbs, both on grip and isokinetic shoulder strength. Conversely, Ahmadi et al. (27) reported a significant strength difference according to dominancy, but it was in favor of the dominant side which exhibited 15% more strength on grip, and from 6.5 to 12.5% on shoulder isokinetic peak torques. Consequently, the difference of limb dominance might have only partially reduced the strength difference between upper-limbs with NTOS and those without. Indeed, the symptomatic sides could have been even more deficient compared to the asymptomatic ones.

Our study is mainly limited due to our criteria of inclusion. Indeed, we recruited patients with NTOS addressed to inpatient rehabilitation because of ineffective outpatient physiotherapy. So, we may have included the most disabled patients preventing us from generalizing these results to all the patients with NTOS.

## Conclusion

Symptomatic upper-limbs of patients with NTOS presented a significant deficit of strength on hand grip, key pinch and shoulder rotators compared to asymptomatic sides. Isometric grip and key pinch strengths assessed with hydraulic dynamometers were moderately correlated to shoulder rotators peak torques. So, hand strength evaluations could provide an overall estimate of the upper-limb weakness in patients with NTOS. Hand hydraulic dynamometers could prove useful during medical consultations or in outpatient management of patients to assess both hand and shoulder weakness. Yet, the correlations remained insufficient for a precise evaluation, especially before surgery or a hospital rehabilitation protocol, for which an accurate and reliable assessment seems preferable. In those last cases, isokinetic shoulder rotators measurement remains the gold standard.

## Data availability statement

The raw data supporting the conclusions of this article will be made available by the authors, without undue reservation.

## Ethics statement

The studies involving human participants were reviewed and approved by Committee of Ethics Comité de Protection des Personnes d'Ile-de-France II (registration: 2019-A02787-50). Written informed consent for participation was not required for this study in accordance with the national legislation and the institutional requirements.

## Author contributions

Conceptualization, project administration, and writing—original draft: AF-C. Formal analysis: AF-C and PD. Investigation: PM, PD, GGad, YB, GGau, GP, and AF-C. Methodology: AF-C, PM, PD, and MD. Resources: GGad and YB. Software: MD. Writing—review and editing: PM, PD, GGad, YB, GGau, GP, and MD. All authors have read and agreed to the submitted version of the manuscript.

## Conflict of interest

The authors declare that the research was conducted in the absence of any commercial or financial relationships that could be construed as a potential conflict of interest.

## Publisher's note

All claims expressed in this article are solely those of the authors and do not necessarily represent those of their affiliated organizations, or those of the publisher, the editors and the reviewers. Any product that may be evaluated in this article, or claim that may be made by its manufacturer, is not guaranteed or endorsed by the publisher.
